# Case report: unravelling the puzzle of unicuspid aortic valve with multimodality imaging

**DOI:** 10.1093/ehjcr/ytae269

**Published:** 2024-05-30

**Authors:** Li Pang, Mark A Colantonio, Jessica Arvon, Bryan Raybuck, Sudarshan Balla

**Affiliations:** Heart and Vascular Institute, West Virginia University, 1 Medical Center Drive, Morgantown, WV 26505, USA; Department of Medicine, West Virginia University, 1 Medical Center Drive, Morgantown, WV 26505, USA; Department of Medicine, West Virginia University, 1 Medical Center Drive, Morgantown, WV 26505, USA; Heart and Vascular Institute, West Virginia University, 1 Medical Center Drive, Morgantown, WV 26505, USA; Heart and Vascular Institute, West Virginia University, 1 Medical Center Drive, Morgantown, WV 26505, USA

**Keywords:** Unicuspid aortic valve, Aortic stenosis, Echocardiography, CMR, MDCT, Case report

## Abstract

**Background:**

Unicuspid aortic valve (UAV) represents a rare congenital anomaly characterized by two subtypes: acommissural unicuspid aortic valve and unicommissural unicuspid aortic valve. Acommissural UAV is often diagnosed and corrected during the neonatal period due to haemodynamic instability. Unicommissural UAV leads to aortic stenosis (AS) in early adulthood. The diagnostic challenge associated with UAV primarily stems from its eccentric orifice opening and valvular calcification, resulting in difficult visualization of the commissures and localization of the orifice plane. This case report aims to demonstrate the unique morphological features of UAV through a comprehensive analysis using multimodality imaging.

**Case summary:**

A 61-year-old woman presented to the emergency department for recurrent episodes of dyspnoea. Severe AS was diagnosed on transthoracic echocardiography (TTE) by Doppler haemodynamic measurement. However, follow-up transesophageal echocardiography (TEE) and CT transcatheter aortic valve replacement showed moderate AS by planimetry. Following this, patient was monitored closely, but her dyspnoea kept worsening. Cardiovascular magnetic resonance (CMR) was performed due to persistent dyspnoea, identifying UAV with eccentric loophole orifice with unicommissural attachment and opposite free leaflet edge. The patient was managed medically.

**Discussion:**

TTE is the test of choice for AS that defines valvular morphology by direct visualization and grades the severity by haemodynamic measurement. However, the accuracy of TTE can be limited by poor acoustic windows and heavy valvular calcification. TEE measures aortic valve area (AVA) by planimetry that requires accurate localization of the AV orifice plane. Similarly, it applies to multi-detector computed tomography (MDCT). While CMR is expensive and mainly available in tertiary centres, it can provide additional information when there is discordance.

**Learning points**

- To learn the morphological features of unicuspid aortic valve exhibited on multimodality imaging.
- To understand the limitations and advantages of each imaging modality to assess unicuspid aortic valve.

## Introduction

Aortic stenosis (AS), defined as narrowing of the aortic valve, is the most common valvular pathology noted in North America.^1^ It can be congenital or degenerative in aetiology. Congenital variants are characterized by the number of cusps in the aortic valve. Bicuspid aortic valve (BAV) is found in 1–2% of the population, and unicuspid aortic valve (UAV) is found in 0.02% of the population. The congenital variants are due to failed tissue separation during embryonic development and predispose to AS.^2,3^

Two variants of UAV, unicuspid acommissural and unicuspid unicommissural, have been described based on absence or presence of commissural attachment to the aortic orifice. Acommissural UAV has a pinhole-like orifice and can present with haemodynamic instability in neonates, prompting immediate surgical correction.^3^ Unicommissural UAV typically progresses to severe AS in early adulthood in the fourth decade of life compared to bicuspid or trileaflet aortic valve morphology. The most common valvular dysfunction in UAV is mixed AS and aortic regurgitation (AR), representing 93%, and isolated AS, representing only 7%. Patients present with dyspnoea, angina, and syncope.^2^

Similar to BAV, there is an association between aortopathy and UAV. The incidence of aortic root or ascending aortic dilatation is reported as 76%.^2^ Other cardiac comorbidities (aortic dissection, patent foramen ovale, coarctation, and hypertrophic cardiomyopathy) have also been reported.^3^ The life expectancy for patients with UAV is comparable to general population after aortic valve surgery. Early detection and management are essential to avoid valve-related morbidity and mortality.^1,4^

## Summary figure

**Figure:**
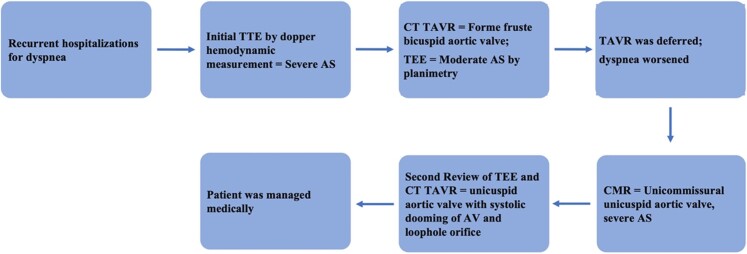


## Case presentation

**Figure 1:**
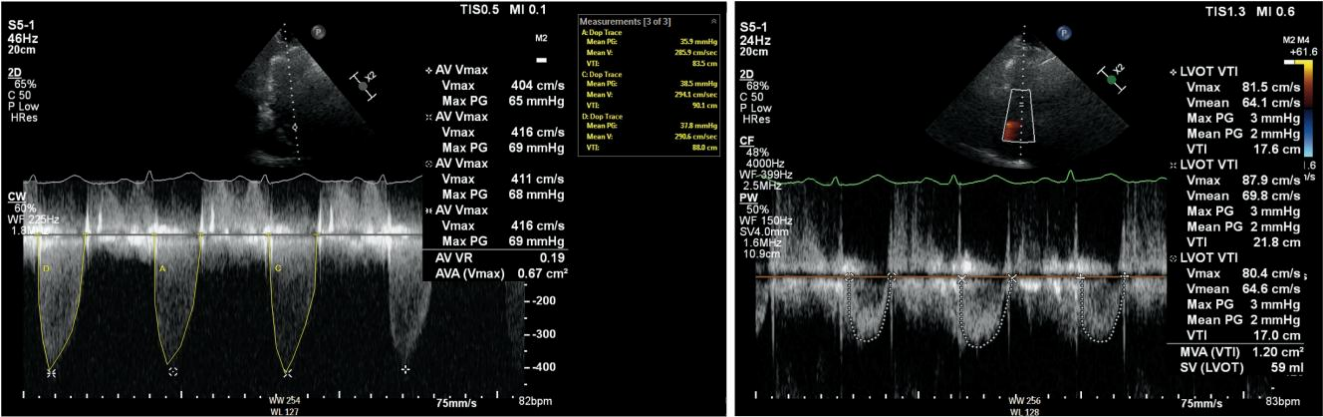
Unicuspid aortic valve TTE three-chamber view (left) shows that Vmax through aortic valve is 4.16 m/s, AV VTI 83.5 cm, 90.1 cm, 88 cm, and average AV VTI 87.2 cm; TTE five-chamber view (right) shows average LVOT VTI 17.6 cm, 21.8 cm, 17 cm, and average LVOT VTI 18.8 cm.

A 61-year-old woman with recurrent hospitalizations for exertional dyspnoea was seen in the emergency department. Her past medical history included paroxysmal atrial fibrillation, chronic obstructive pulmonary disease, hypertension, hyperlipidaemia, and obesity. Patient had 2/6 systolic murmur radiating to the carotid arteries. Transthoracic echocardiography (TTE) (*Figure 1*) revealed severe AS with mild AR and left ventricular ejection fraction (LVEF) 60%, stroke volume index (SVI) 39 mL/m^2^, aortic valve (AV) mean pressure gradient (mPG) 43 mmHg, AV peak velocity (PkV) 416 cm/s, aortic valve area (AVA) by velocity time integral (VTI) 0.89 cm^2^, dimensionless velocity index (DVI) 0.22, and LVOT diameter 2.3 cm. Previously, her TTEs (Supplementary material online, *Videos 1* and *2*) were technically difficult studies with poor cardiac windows. The diagnosis of severe AS was based on haemodynamic gradients and AVA calculated via continuity equation (CE). Invasive coronary angiogram showed non-obstructive coronary artery disease.

Heart Team evaluated the patient for aortic valve replacement (AVR). Patient was deemed a poor surgical candidate due to comorbidities and elevated Society of Thoracic Surgeon (STS) score; transcatheter aortic valve replacement (TAVR) was recommended. Patient underwent transesophageal echocardiography (TEE) (Supplementary material online, *Video 3*), which showed AVA 2.2 cm^2^ by planimetry, AVA by VTI 0.89 cm^2^ (PkV 3.1 m/s, mPG 25 mmHg, DVI 0.28, LVOT diameter 2.0 cm). The CT TAVR revealed a forme fruste bicuspid valve with a calcium score of 377 AU and valve area of 1.5 cm^2^ by planimetry (35% phase), LVOT diameter 2.55 cm × 2.16 cm, LVOT area 4.27 cm^2^, and small iliofemoral arteries bilaterally (<6 mm).

**Figure 2:**
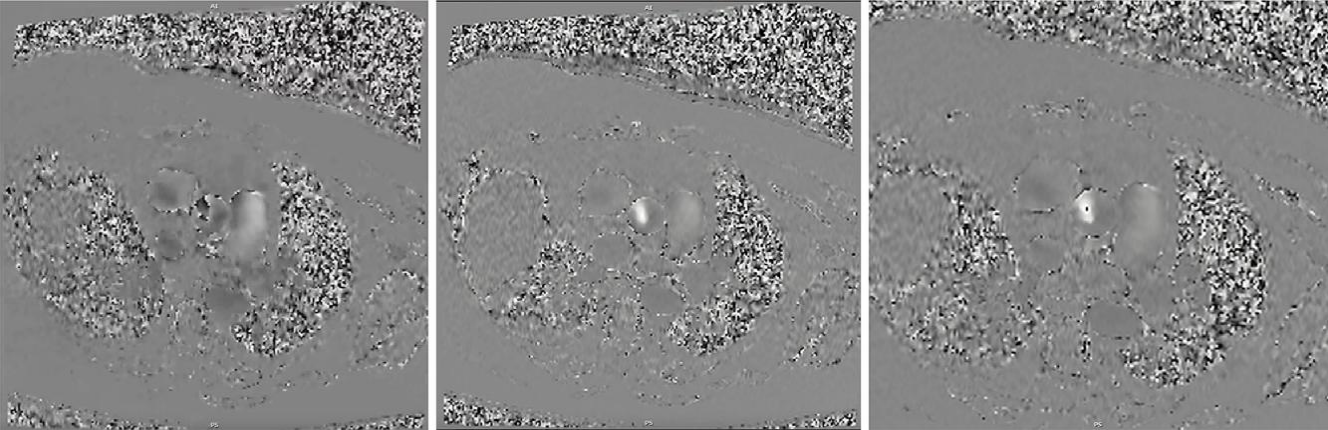
Phase contrast CMR at different VENC settings shows aliasing at 3.5 m/s (left), complete resolution of aliasing at 4.5 m/s (middle), and minimal aliasing at 4.0 m/s (right).

**Figure 3:**
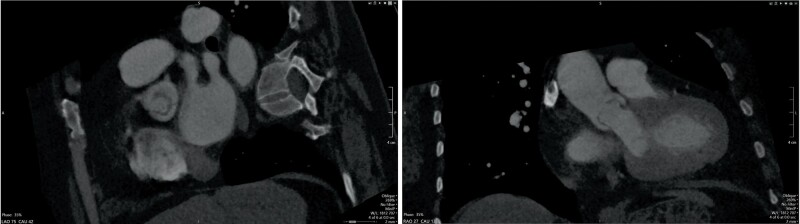
CT TAVR (35% phase) shows aortic valve opening appears eccentric, loophole-shaped on the short axis view (left); systolic dooming of the aortic valve leaflets on the long axis view (right).

TAVR was deferred as the aortic stenosis appeared insignificant based on the above data, however, patient’s dyspnoea continued to worsen. Due to worsening symptoms and prior inconsistent results, cardiovascular magnetic resonance (CMR) (Supplementary material online, *Videos 4*–*7*) was performed, which demonstrated a unicommissural UAV with AVA 0.63 cm^2^ by planimetry. Velocity encoded (VENC) sequences were obtained at different velocities with VENC of 4 m/s showing mild aliasing that resolved when VENC was increased to 4.5 m/s, confirming the diagnosis of high gradient severe AS (*Figure 2*). The patient was deemed high risk for TAVR due to inadequate vascular access, underlying severe chronic obstructive pulmonary disease with oxygen and steroid dependence. Upon second review of the CT TAVR (*Figure 3*), unicuspid valve morphology was noted with eccentric, loophole-shaped orifice on short axis view and systolic dooming of the aortic valve leaflets on the long axis view. The AVA calculated by VTI from TTE and LVOT area from CT was 0.92 cm^2^. On further review of prior TEE (Supplementary material online, *Video 3*), the dooming of aortic valve leaflets during systole was noted, indicative of incomplete separation of aortic valve leaflets. This was suggestive of UAV. There was no 3D dataset of the aortic valve at time of TEE for reconciliation. Unfortunately, patient continues to have dyspnoea, being managed with diuretics.

## Discussion

Two-dimensional echocardiography remains the diagnostic imaging modality of choice for AS. As per American Society Echocardiography (ASE) and European Society of Cardiology (ESC) Guidelines, the CE is the method of choice to measure AVA. The aortic valve morphology is illustrated by direct visualization of the aortic valve.^5^

However, diagnosing UAV can be challenging. In prior reports, UAV was diagnosed by TTE (14–25%) and by TEE (69–75%) due to the prevalence of concomitant valvular calcification and the unfamiliar morphological features of UAV.^1^ Common echocardiographic findings in UAV include single commissural attachment zones; rounded, leaflet-free edge opposite from commissural attachment zone; eccentric valvular orifice; and mean transvalvular gradient > 15 mmHg at age < 20 years.^6^ In our case, AVA was initially reported as mild stenosis by 2D TEE planimetry, contradictory to the Doppler haemodynamic measurement. Conflicting data between AVA by planimetry and CE should raise suspicion for measurement error, valvular variants, and subvalvular or supravalvular obstruction. We suspect that the AVA was overestimated by 2D TEE planimetry due to the inaccurate plane of the leaflet tips from the eccentric orifice opening.

The underutilization of 3D TEE is a limitation in our case. 3D TEE is recommended as part of a standard examination of valvular diseases and intracardiac devices.^7,8^ Three-dimensional TEE with multiplanar reconstruction allows accurate localization of true orifice opening and identify the narrowest orifice for true estimation of anatomical AVA. The 3D planimetered LVOT area can be used to calculate AVA using the continuity equation, avoiding the assumption of a circular LVOT used in 2D linear measurement.^9^ Additionally, TEE with transillumination technique available on some equipment has been described to improve visualization of the unicommissure on 3D TEE.^10^ It is crucial to use 3D TEE to comprehensively assess the aortic root complex, and to guide procedures on the aortic valve.^8^

The aortic valve calcium score of 377 AU from CT TAVR leads us to underestimate severity of AS. CT aortic valve calcium score (>1300 for women, >2000 for men) has high diagnostic value in detecting severe AS, but it is not validated for UAV.^5^ ECG-gated cardiac CT planimetry tend to overestimate AVA.^11^ CMR can be a reliable alternative to measure AVA by direct planimetry on CineMR images and CE with transvalvular aortic flow through phase contrast MRI when patients have poor acoustic windows.^11–13^ In our case, CMR located the UAV commissure and characterized the direction of jet and localization of flow acceleration.

TEE, CT, and CMR are recommended when evaluating associated aortopathy and other congenital anomalies with UAV (patent ductus arteriosus, ventricular septal defect, coarctation of aorta, and coronary artery anomalies).^14^ The test of choice can vary based on the clinical need to assess valvular function, and patients’ clinical condition including haemodynamic stability, renal function, and patient’s tolerance. Our patient underwent multimodality imaging to establish the diagnosis of UAV with isolated severe AS with mild aortic insufficiency.

## Conclusion

Unicuspid aortic valve is a rare valve variant and could be misdiagnosed. Our case highlights the distinctive imaging features of UAV and the advantages and limitations of each imaging modality.

## Lead author biography


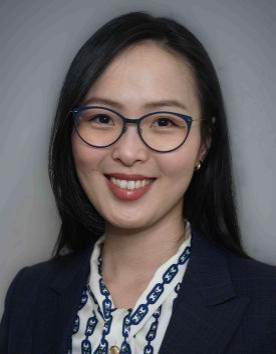


Dr Li Pang graduated in 2018 from Tongji University School of Medicine, Shanghai, China. She completed her internal medicine residency training programme at Jacobi Hospital affiliated with Albert Einstein College of Medicine. She is currently a Cardiology fellow at West Virginia University, WV, USA. Her main areas of interest are cardiovascular imaging and interventional cardiology.

## Supplementary Material

ytae269_Supplementary_Data

## Data Availability

The data underlying this article are available in the article and in its online Supplementary material.

## Supplementary material

**Consent:** Consent has been obtained from the patient for participation and use of data in this case report. The patient has been informed that consent does not remove the patient’s right to privacy and the patient has been informed that he/she may revoke consent at any time prior to publication. The patient was informed that use of this case report may be published, meaning that it may be printable online. The patient was notified that he/she will not expect financial benefit from publication. The authors confirm that written consent for submission and publication of this case report including the images and associated text has been obtained from the patient in line with COPE guidance.

**Funding:** None.
